# Supplementary material for: The acceptability of the AMBITION-cm treatment regimen for HIV-associated cryptococcal meningitis: Findings from a qualitative methods study of participants and researchers in Botswana and Uganda
Source: PLoS Negl Trop Dis. 2022 Oct 24;16(10):e0010825. doi: 10.1371/journal.pntd.0010825 (PMC9632910; doi:10.1371/journal.pntd.0010825)
Supplement: S1 File — (PDF) [file pntd.0010825.s001.pdf]

The Lived Experience Of Participants in an African RandomiseD controlled trial (LEOPARD)

Participant In-depth Interview Schedule

Note: This is purely a guide for a semi-structured interview and is not a rigid script. The interview should attempt to cover the key themes of enquiry outlined below but the participant should be able to steer the conversation and deviate from these themes if desired.

Introduction:

- General purpose and overview of the study
- Aims of interview
- Why the participant's cooperation is important
- Assurance of confidentiality
- What will happen with the collected information
- Any questions?
- Consent

'The aim of this exercise and series of questions is to understand a little about you and to hear your experience of the trial process from before you were recruited, the consent process, and throughout the trial itself'

Demographics and Background

- Age
- Gender
- Occupation

'At this point I would like you to consider drawing your experience in the trial as a timeline onto this piece of paper. I would like to know how you experienced each of the parts of the trial, one after the other, from just before you joined the trial until today.'

The participant can decline this approach if they wish. If they do want to draw a timeline let them direct the conversation and try to understand their recollection of events. Use prompts to ask follow-up questions as suggested below.

Before the study

- Previous experience with clinical trials, if any
- Previously held perceptions of clinical trials
- General health
- Circumstances in which participant became unwell
- How dis/orientated they were, their recollection of events
- The admission to the hospital, including experience of diagnostic lumbar puncture
- Diagnosis of cryptococcal meningitis and any other illness

Recruitment

- Experience of being approached by the team

- First impressions of the clinical trial
- Thoughts on the participant information sheet

Consent

- How did they decide
- What was their motivation and what were their main concerns
- How long did it take to decide
- Did they feel under pressure to consent and if so, by whom
- With whom did they decide
- Did someone decide on their behalf and if so, what is their recollection of that and how did they feel both around that time and now
- When completing the form did they feel that they knew what they were signing up for
- Is there any way this process could have been improved

Within the trial (inpatient)

- Was anything different after they entered the trial compared to before
- What did they think about the nature, number and frequency of the procedures they had e.g. blood tests and lumbar punctures
- What did they think about the drugs they were receiving particularly the night time doses
- Are there any specific experiences whilst in hospital they would like to discuss
- Were they confused by what was going on at any point
- How was the communication and care from the trial team

Within the trial (outpatient)

- How was the outpatient clinic and did you have any concerns (such as confidentiality, security, cleanliness)
- How was your experience of those outpatient visits
- Did they miss any appointments during the trial and if so, why
- At any time did they consider leaving the trial and if so, why
- What did they think about the transport reimbursement, was it enough, did it play a role in encouraging them to attend outpatient visits
- How did they feel being asked the health economics questions
- Can you summarise the AMBITION trial

For participants that were confused, ask these questions at appropriate moments

- Did they understand what was happening
- If not, when did they begin to understand what was happening
- Did their confusion resolve all at once or did it come and go
- When they were informed they were in a clinical trial, what were their thoughts
- Who provided consent for them when they were confused, have they discussed this with the person/people and how do they feel about this now

For participants that have completed the study, ask these questions at appropriate moments

- How do they feel now they have left the trial
- Have they been back to their usual care provider and if so, how was that experience

- What would they like to have seen done differently within the course of the trial
- If they were approached to take part in a clinical trial in the future what would they do and why

Closing:

Is there anything else you think is important that we have not talked about?

- Summarise
- Thank participant
- Provide contacts to participant

Second interview

A second interview will take place after the participant has exited the AMBITION study. During the second interview, spend time reviewing the information that was captured in the first and asking the participant if they have changed how they feel since exiting the study.

Any aspects of this interview schedule that were not captured in the first interview can be addressed in the second.

The Lived Experience Of Participants in an African RandomiseD controlled trial (LEOPARD)

Next-of-kin In-depth Interview Schedule

Note: This is purely a guide for a semi-structured interview and is not a rigid script. The interview should attempt to cover the key themes of enquiry outlined below but the participant should be able to steer the conversation and deviate from these themes if desired.

Introduction:

- General purpose and overview of the study
- Aims of interview
- Why the participant's cooperation is important
- Assurance of confidentiality
- What will happen with the collected information
- Any questions?
- Consent

'The aim of this exercise and series of questions is to understand a little about you and to hear your experience as the next-of-kin of someone who was recruited into the AMBITION trial. We are interested to hear your experience of the trial process for your loved one from before they were recruited, the consent process, and throughout the trial itself'

Demographics and Background

- Age
- Gender
- Occupation

'At this point I would like you to consider drawing your experience with the trial as a timeline onto this piece of paper. I would like to know how you experienced each of the parts of the trial, one after the other, from just before you were aware of the trial until today.'

The next-of-kin participant can decline this approach if they wish. If they do want to draw a timeline let them direct the conversation and try to understand their recollection of events. Use prompts to ask follow-up questions as suggested below.

Before the study

- Previous experience with clinical trials, if any
- Previously held perceptions of clinical trials
- Circumstances in which the trial participant became unwell
- How dis/orientated they felt to be at the time
- The admission to the hospital, including experience of diagnostic lumbar puncture
- How and if they were informed of the diagnosis of cryptococcal meningitis and any other illness

Recruitment

- Experience of being approached by the team
- First impressions of the clinical trial
- Thoughts on the participant information sheet

Consent (Next-of-kin perspective)
- How did they decide
- What was their motivation and what were their main concerns
- How long did it take to decide
- Did they feel under pressure to consent and if so, by whom
- With whom did they decide
- When completing the form did they feel that they knew what they were signing their loved one up for
- Is there any way this process could have been improved

Consent (Participant perspective)
- Did they think their loved one understood what was happening
- If not, when did they begin to understand what was happening
- Did their confusion resolve all at once or did it come and go
- When they were informed they were in a clinical trial, were they part of the re-consent process
- Have they ever discussed this issue of consent with their loved one and if so would they be willing to share this discussion with the researcher

Within the trial (inpatient)
- Was anything different after the participant entered the trial compared to before
- What did they think about the nature, number and frequency of the procedures their loved one had e.g. blood tests and lumbar punctures
- What did they think about the drugs they were receiving particularly the night time doses
- Are there any specific experiences whilst in hospital they would like to discuss
- Were they confused by what was going on at any point
- How was the communication and care from the trial team
- Did they feel that they were involved in the trial process

For the next-of-kin that accompanied the participant to outpatient appointments, ask these questions at appropriate moments
- How was the outpatient clinic and did you have any concerns (such as confidentiality, security, cleanliness)
- How was your experience of those outpatient visits
- Did your loved one miss any appointments during the trial and if so, why
- At any time did they consider removing their loved one from the trial and if so, why
- At any time did their loved one consider removing themselves from the trial and if so, why
- What did they think about the transport reimbursement, was it enough, did it play a role in encouraging their loved one to attend outpatient visits and for them to accompany them
- Can they summarise the AMBITION trial

Reflections on the trial

- How do they feel about the trial in general
- What would they like to have seen done differently within the course of the trial
- If they were approached to take part in a clinical trial in the future what would they do and why

Closing:

Is there anything else you think is important that we have not talked about?

- Summarise
- Thank participant
- Provide contacts to participant

The Lived Experience Of Participants in an African RandomiseD controlled trial (LEOPARD)

Researcher In-depth Interview Schedule

Note: This is purely a guide for a semi-structured interview and is not a rigid script. The interview should attempt to cover the key themes of enquiry outlined below but the participant should be able to steer the conversation and deviate from these themes if desired.

Introduction:

- General purpose and overview of the study
- Aims of interview
- Why the participant's cooperation is important
- Assurance of confidentiality
- What will happen with the collected information
- Any questions?
- Consent

'The aim of the first series of questions is to contextualise you within the Ambition study and the clinical research community'

Demographics and Background:

- Job title and role
- Institution and number of years there
- Training / qualifications and their locations
- Research posts previously held and their locations

The Ambition study:

- How became involved
- Current role and responsibilities
- Level of engagement with trial participants

Previous research experience:

- Background of working with participants of other trials
- With individuals within Ambition and/or not affiliated
- In other institutions
- In transnational research partnerships

'Drawing predominantly on your current experience within the Ambition study but also from your previous work (if any) please can you share your thoughts on the following:'

Trial participant experience:

- General impressions of how participants experience a trial
- What you and fellow researchers are good at
- What you are not so good at
- Experience of evaluating trial participant experience

- Suggestions for improvements

Specifically aim to focus on: the consent process, recruiting participants with impaired consciousness and the death of participants, prompting if required. Probing to draw on specific examples to elicit narratives.
‘Do you think that the issues you have brought up are specific to your hospital / institution / city / country. Where else can you see they do / may occur?’

Transnational research partnerships:

- Understanding of the EDCTP and how it works
- Perceived benefits of such an approach
- Any shortcomings
- Capacity building
- Ownership
- Impact on the global research agenda
- Any suggestions for improvement

‘Do you think that the issues you have brought up are specific to your hospital / institution / city / country. Where else can you see they do / may occur?’

Closing:

Is there anything else you think is important that we have not talked about?

- Summarise
- Thank participant
- Provide contacts to participant
