# Supplementary material for: The acceptability of the AMBITION-cm treatment regimen for HIV-associated cryptococcal meningitis: Findings from a qualitative methods study of participants and researchers in Botswana and Uganda
Source: PLoS Negl Trop Dis. 2022 Oct 24;16(10):e0010825. doi: 10.1371/journal.pntd.0010825 (PMC9632910; doi:10.1371/journal.pntd.0010825)
Supplement: Attachment — Submitted filename: Response to Reviewers.docx [file pntd.0010825.s002.docx]

5^th^ September 2022

Dear Dr Xue,

**The acceptability of the AMBITION-cm treatment regimen for HIV-associated cryptococcal meningitis: findings from a qualitative methods study of participants and researchers in Botswana and Uganda**

**REF: PNTD-D-22-00798**

We would like to thank you and the reviewers for the comments on this manuscript. We hugely appreciate you offering up your time and expertise to appraise the paper and provide suggestions for improvement.

We are happy with the positive comments and recommendations of the reviewers and have addressed each of their comments. We believe that the submission is now stronger and clearer as a result.

In this response letter we will respond below to each reviewer’s comments one-by-one. The page numbers we provide pertain to the tracked changes version of the paper.

**Reviewer One**

*The manuscript by Lawrence et al describes results of a qualitative follow up study to the Ambisome Therapy Induction Optimization clinical trail, aimed at investigating the acceptance of the treatment regiments by patients, their caregivers and healthcare workers participating in the clinical trial. The study is based on the interviews and direct observations. The manuscript is well written, the results are important.*

AU: Many thanks for this positive overview of the manuscript.

*In my opinion, the paper will benefit from providing more concrete data in support the observed tendencies. A few suggestions are below:*

*(1) consider including the interview questions into supplemental materials (were the same interview questions used in all study sites?)*

Thank you for this comment. We used three interview schedules, tailored to the category of participant (trial participant, surrogate decision maker, and researcher) and the same interview schedule was used across both sites. We have included these as supplementary material and mentioned this in the methods on Page 8 Line 176. We conducted in-depth interviews within this study. This approach provides the opportunity for the conversation to flow, to ask follow-up questions, probe for additional information, and circle back to key questions later. In general, this approach provides richer, more in-depth data than structured interviews. We have added the above wording to Page 8 Line 155.

*(2) Please describe steps taken to ensure that the interviewer was not inadvertently influencing the participant’s opinion in favor of one arm versus the other.*

AU: It would not be ethical to knowingly influence the participants’ response however, as you have said, inadvertent influence can occur. To try and minimise this we conducted in-depth interview which used open-ended questions and allowed participants to speak freely. We also recruited social scientists who were separate from the AMBITION-cm trial to collect data from trial participants and SDMs in Gaborone and Kampala. As this manuscript also highlights the shortcomings of the AMBITION-cm regimen we believe this demonstrates that participants were able to speak freely about the pros and cons of both arms. Despite this, we acknowledged the potential for desirability bias and Hawthorne effect in the limitations section of the discussion and we have expanded on this further in the revised manuscript on Page 17 Line 374 which now reads:

*‘Finally, we acknowledge the positionality of the research team, including DSL as the Chief Investigator of this study and the lead clinician of the AMBITION-cm trial, and how this may have resulted in some desirability bias and a Hawthorne effect during data collection. We aimed to overcome this by forming a research group including social scientists external to the trial who collected the data from participants and SDMs in Gaborone and Kampala.’*

*(3) If possible, consider showing the participants’ demographic information at each study site, including the language, in which the interview was conducted. These data can be organized in a table or graph format*

AU: Thank you for this suggestion. We have summarised the participants in a newly produced Table 1. As we have mentioned in the methods, we did not present demographic data on the researchers due to the small number of eligible individuals and the need to maintain confidentiality and anonymity.

*(4) Instead of using subjective terms like “general preference”, “most participants”, “clear preference”, etc., please consider including the actual numbers of participants favouring one arm of the trial over the other… Is it possible to assign numbers to these terms?*

AU: When adopting an in-depth approach, as we have here, it is not typical to present opinions in numerical form. Within our limitations we explain that we adopted a purposive recruitment strategy and that the results are not intended to be fully representative or generalisable. As we have discussed, particularly from the perspective of trial participants, the severity of their illness and the acuity of their unfolding health emergency made it difficult to disentangle the different aspects of their care. We do not feel it would therefore be useful or representative to present these data as proportions or percentages and instead would request that we use this same language which is consistent with similar studies conducted within this discipline.

**Reviewer Two**

The conclusion from the paper is in line with known knowledge evidenced by the WHO 2022 guideline and the paper (ref 21) the authors cited. The extra information the authors provided was evidence for acceptability of Ambisome regimen I am not sure if this is enough data for a full research article. The sample size is very small

AU: Thank you for your comments and overview of the paper. These are the only data that have explored this important aspect of the AMBITON-cm treatment regimen. Upon request, this analysis was presented in confidence to the WHO Guideline Writing Committee in February 2022 and is the foundation for the wording you have mentioned in the new guideline. We consider this analysis to be an important contribution to the evidence base around this treatment regimen.

In terms of sample size, we recruited in-line with our a-priori sample size targets. When compared with similar qualitative methods studies this is quite a large sample size, having recruited 89 individuals^1-4^. As discussed within the results and discussion, we found broad consensus with our conclusions which we feel indicate good levels of generalisability however we do acknowledge within our limitations that the results are not intended to be fully representative or generalisable to every context.

**General Editorial Comments**

AU: We have uploaded the figures to PACE. This was a purely qualitative study and data are available upon reasonable request to the corresponding author. Finally, we have reviewed the reference list to ensure that it is complete and correct. As a result, we have updated the epidemiological data and Reference 1 on Page 5 Line 76 following the very recent publication of updated Global Burden of Disease estimates. We have also updated reference 22 as the revised WHO guidelines have now been published.

Many thanks again. We look forward to receiving your additional feedback.

On behalf of all authors,

Dr David Lawrence

Clinical Research Physician

The London School of Hygiene and Tropical Medicine

The Botswana Harvard AIDS Institute Partnership

**References:**

1.	Prout HC, Barham A, Bongard E, et al. Patient understanding and acceptability of an early lung cancer diagnosis trial: a qualitative study. *Trials* 2018; **19**(1): 419.

2.	Atujuna M, Newman PA, Wallace M, et al. Contexts of vulnerability and the acceptability of new biomedical HIV prevention technologies among key populations in South Africa: A qualitative study. *PloS one* 2018; **13**(2): e0191251.

3.	Pell C, Meñaca A, Chatio S, Hodgson A, Tagbor H, Pool R. The acceptability of intermittent screening and treatment versus intermittent preventive treatment during pregnancy: results from a qualitative study in Northern Ghana. *Malaria journal* 2014; **13**(1): 432.

4.	Alhassan Y, Twimukye A, Malaba T, et al. Community acceptability of dolutegravir-based HIV treatment in women: a qualitative study in South Africa and Uganda. *BMC public health* 2020; **20**(1): 1883.
